# A central-acting connexin inhibitor, INI-0602, prevents high-fat diet-induced feeding pattern disturbances and obesity in mice

**DOI:** 10.1186/s13041-018-0372-9

**Published:** 2018-05-24

**Authors:** Tsutomu Sasaki, Rika Numano, Hiromi Yokota-Hashimoto, Sho Matsui, Naobumi Kimura, Hideyuki Takeuchi, Tadahiro Kitamura

**Affiliations:** 1 Laboratory of Metabolic Signaling, Institute for Molecular and Cellular Regulation, Gunma University, 3-39-15 Showa-machi, Maebashi, Gunma 371-8512 Japan; 2 Department of Environmental and Life Sciences, Toyohashi University of Technology, 1-1 Hibarigaoka Tempaku-cho, Toyohashi, 441-8580 Japan; 3 Electronics-Interdisciplinary Research Institute (EIIRIS), Toyohashi University of Technology, 1-1 Hibarigaoka Tempaku-cho, Toyohashi, 441-8580 Japan; 4 Department of Neurology and Stroke Medicine, Yokohama City University Graduate School of Medicine, 3-9, Fukuura, Kanazawa-ku, Yokohama, 236-0004 Japan

**Keywords:** Diet-induced obesity, Feeding clock, Feeding rhythm, Gap junction, Hyperphagia, Hypothalamic inflammation, Microglia, Saturated fatty acids

## Abstract

A high-fat diet (HFD) causes obesity by promoting excessive energy intake, and simultaneously, by disturbing the timing of energy intake. Restoring the feeding pattern is sufficient to prevent HFD-induced obesity in mice. However, the molecular mechanism(s) underlying HFD-induced feeding pattern disturbances remain elusive. Saturated fatty acids activate microglia and cause hypothalamic inflammation. Activated microglia cause neuroinflammation, which spreads via inflammatory cytokines and gap-junction hemichannels. However, the role of gap-junction hemichannels in HFD-induced obesity remains unaddressed. We used a novel, central-acting connexin inhibitor, INI-0602, which has high affinity for gap junction hemichannels and does not affect the induction of inflammatory cytokines. We analyzed ad libitum feeding behavior and locomotor activity in mice that were fed normal chow (NC), a HFD with elevated saturated fatty acids (SFAs), or a HFD with very high SFAs. We found that HFD feeding induced acute hyperphagia, mainly during the light cycle. Feeding pattern disturbances were more pronounced in mice that consumed the HFD with very high SFAs than in mice that consumed the HFD with elevated SFAs. When INI-0602 was administered before the HFD was introduced, it blocked the feeding pattern disturbance, but not locomotor activity disturbances; moreover, it prevented subsequent diet-induced obesity. However, when INI-0602 was administered after the HFD had disturbed the feeding pattern, it failed to restore the normal feeding pattern. Therefore, we propose that SFAs in HFDs played a major role in disrupting feeding patterns in mice. Moreover, the feeding pattern disturbance required the function of central, gap junction hemichannels at the initiation of a HFD. However, altering hemichannel function after the feeding pattern disturbance was established had no effect. Thus, preventing the occurrence of a feeding pattern disturbance by blocking the hemichannel pathway was associated with the prevention of the HFD-induced obesity in mice.

**Electronic supplementary material:**

The online version of this article (10.1186/s13041-018-0372-9) contains supplementary material, which is available to authorized users.

## Introduction

Food consumption is characterized by three aspects: what, when, and how much. The content, the timing, and the size of meals are important factors in dietary effects on health [1]. Ingesting the same food at different times of the day has different consequences on health, because systemic metabolic efficiency fluctuates over the course of the day [2]. In humans, food intake at later times in the circadian rhythm was associated with increased adiposity, independent of the content or amount of food intake [3]. Indeed, among humans enrolled in weight-loss programs, those that ingested more calories earlier in the day lost more weight and showed more improvement in metabolic markers compared to those that ingested more calories late in the day [4, 5]. Therefore, eating at the correct time is beneficial for health.

High fat diets (HFDs, or “what” you eat) promote obesity. First, HFDs cause excessive energy intake (affecting “how much” you eat); second, HFDs disturb various rhythms, such as feeding (affecting “when” you eat), locomotor activity, and metabolism [6–8]. A lack of coordination in feeding, activity, and metabolism can desynchronize energy intake and expenditure. Correcting the feeding schedule was sufficient to prevent HFD-induced obesity in mice, even without changing caloric intake [9, 10]. Accumulating evidence has emphasized the importance of feeding patterns (when you eat) in maintaining energy balance and health. To facilitate healthy eating behavior, it is essential to understand the mechanisms that drive ad libitum feeding patterns. However, it remains elusive how feeding patterns are regulated physiologically and how they become disturbed with a HFD.

Some studies have shown that the excessive energy intake associated with HFD ingestion is mediated by activated microglia, which cause hypothalamic inflammation [11, 12]. HFDs contain saturated long-chain fatty acids (SFAs), such as palmitic acid (C16:0) and stearic acid (C18:0), which accumulate in the hypothalamus [13]. This accumulation activates toll-like receptor 4 signaling, which induces the transcription factor, NF-κB, to upregulate inflammatory cytokine production (e.g., TNF-α) [12, 14]. The acute microglial activation induced by HFD feeding is restricted to the arcuate nucleus (ARC) of the hypothalamus [11, 12], which is the primary center for the homeostatic control of body weight [15]. The resulting hypothalamic inflammation causes resistance to the central anorexigenic signals of leptin and insulin. This resistance disrupts the homeostatic regulation of feeding and body weight and leads to hyperphagia and weight gain [12, 16]. However, it remains unclear what role hypothalamic inflammation plays in feeding patterns. Importantly, inflammatory cytokines are not the only pathway for spreading neuroinflammation. Indeed, neuroinflammation mediated by activated microglia is known to spread through two pathways: the inflammatory cytokine pathway (mediated by TNF-α, IL-1β, etc.) and the gap-junction hemichannel pathway [17]. The hemichannel pathway was shown to play a major causative role in promoting neuronal damage in neurodegenerative diseases [17].

INI-0602 is a central-acting, pan-connexin inhibitor with a higher affinity for hemichannels than for gap junctions [18]. INI-0602 blocks only microglial release of small molecules (such as glutamate) through hemichannels, without attenuating acute inflammatory cytokine induction [18]. We previously reported that inhibiting this pathway with INI-0602 did not affect inflammatory cytokines; nevertheless, it suppressed disease progression in mouse models of amyotrophic lateral sclerosis and Alzheimer’s disease by blocking glutamate release into the extracellular space [18]. Importantly, INI-0602 was designed to target the central nervous system (CNS); the CNS redox system oxidizes the dihyropyridine moiety of the pro-drug, which results in the drug becoming trapped within the CNS [19]. Therefore, INI-0602 accumulates in the CNS, including the hypothalamus, although it is rapidly cleared from the circulation and peripheral tissues [18]. Due to this pharmacodynamic property, INI-0602 demonstrated no systemic side effects in mice, even after chronic administration for 5 months [18]. Therefore, INI-0602 is an ideal tool for investigating whether the gap junction hemichannel pathway plays a role in HFD-induced hypothalamic inflammation.

Although many investigators have extensively studied the importance of inflammatory cytokine signaling in the pathogenesis of obesity, the role of the gap junction hemichannel pathway in HFD-induced obesity has not been addressed. Therefore, in this study, we investigated whether treating HFD-fed mice with INI-0602 would be sufficient for preventing HFD-induced obesity and the associated feeding pattern disturbances. By monitoring feeding patterns in detail, we found that INI-0602 prevented HFD-induced feeding pattern disturbances, characterized by excessive feeding during the light cycle. This effect was associated with the prevention of the HFD-induced obesity in mice.

## Results

### INI-0602 prevented HFD-induced obesity in mice

**Fig. 1:**
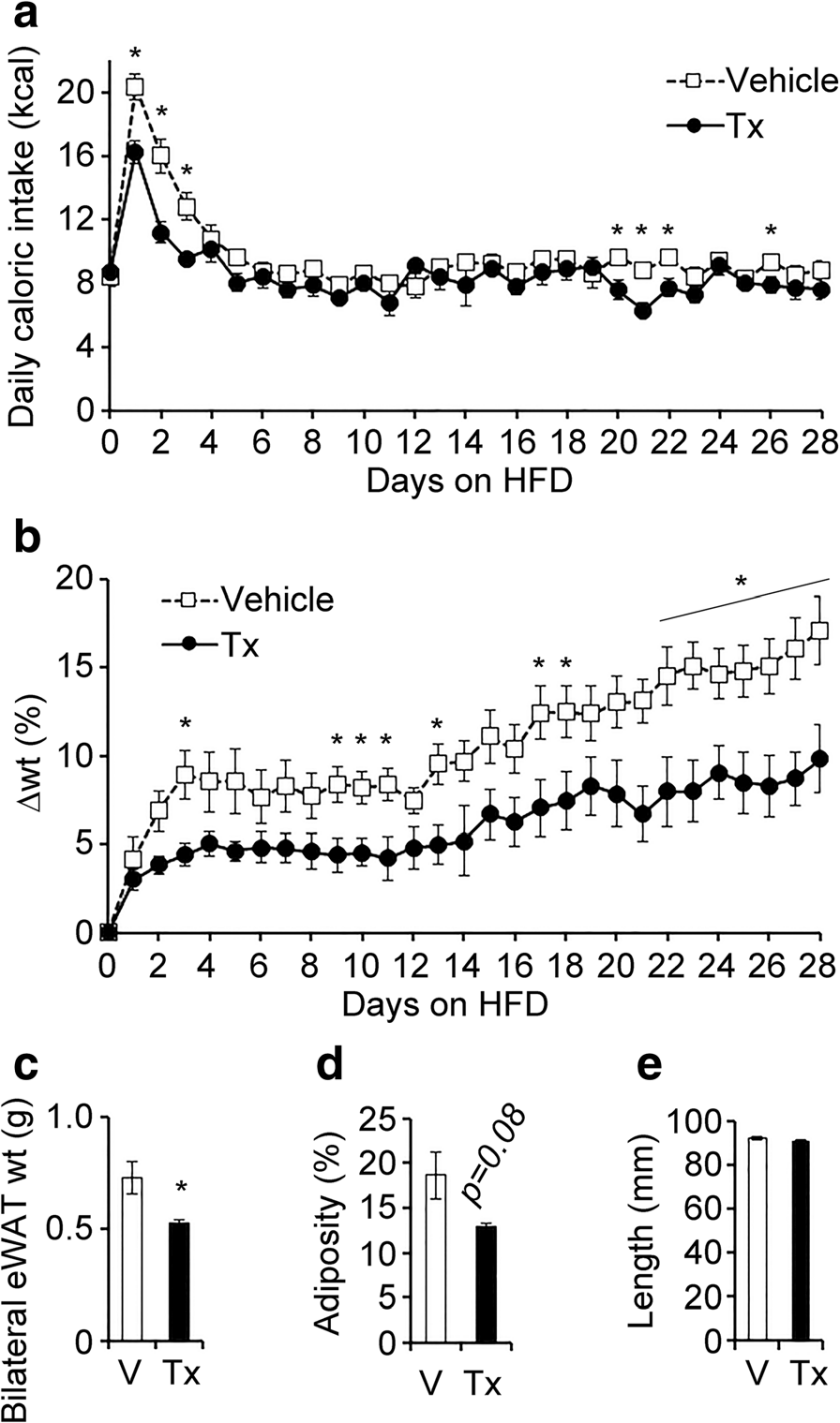
INI-0602 prevented HFD-induced obesity in mice. (**a** and **b**) Mice received intraperitoneal administrations of vehicle (V, *n* = 6, white squares with dashed line) or INI-0602 (Tx, *n* = 5, black circles with solid line) every other day for 28 days during a HFD. (**a**) Daily caloric intake and (**b**) daily changes in body weight are shown, starting from the day of the first injection (day 0). At the end of the 4-week study, we measured (**c**) bilateral epididymal white adipose tissue (eWAT) weight, (**d**) adiposity (based on computed tomography), and (**e**) body length (from snout to anus). Data are the means ± s.e.m. Statistical significance was determined with the Student’s *t*-test, for comparisons between the two groups at each time point. *P < 0.05. Abbreviations: *A.U.* arbitrary unit, *eWAT*epididymal white adipose tissue, *HFD* high-fat diet, *wt* weight

To test whether the central-acting gap junction hemichannel pathway inhibitor, INI-0602, could prevent HFD-induced obesity, we first fed male C57BL/6 J mice HFD60 feed (HFD rich in SFAs) for 4 weeks, starting at 12 weeks of age. INI-0602 or vehicle was intraperitoneally injected at the beginning of the light cycle (0800 h) every other day. Also, at 0800, mice were switched from normal chow (NC) to HFD60 feed. We found that, in the initial 3 days after the diet was switched from NC to HFD, vehicle-treated mice began to exhibit hyperphagia, but INI-0602 treatment significantly suppressed this hyperphagic response (Fig. 1a). Switching the diet from NC to HFD is known to cause hypothalamic inflammation in two phases. The acute inflammation phase occurs within 1–3 days, and the chronic inflammation phase occurs after 2 weeks [11]. Although caloric intake remained stable after the first week, mice started to gain weight after 2 weeks in both groups, which corresponded to the timing of the chronic hypothalamic inflammation phase (Fig. 1a-b).

At the end of the 4-week study, the INI-0602–treated group gained significantly less weight compared to the vehicle-treated group (Fig. 1b). The treatment group had significantly lower epididymal fat weight (Fig. 1c) and tended to exhibit less adiposity than controls (Fig. 1d). The two groups were not different in body length (Fig. 1e); this indicated that the suppression of weight gain was not due to a suppression of normal growth, but due to reduced adiposity. Microglial activation was assessed with histological anti-Iba-1 staining of tissue sections prepared from the ARC at the end of the 4-week study (at this point, the initial activation of microglia by HFD has subsided and turned into the chronic inflammation phase). The two groups showed similar numbers of cells stained with anti-Iba-1 and similar Iba-1–positive areas within the ARC (Additional file 1: Figure S1a-c). Therefore, INI-0602 prevented HFD-induced obesity and attenuated the initial hyperphagic response that occurred with the diet switch from NC to HFD.

### HFD with elevated SFAs caused feeding pattern disturbances by increasing intake during the light cycle

We noted that INI-0602 was more effective in suppressing HFD-induced hyperphagia during the first several days after the diet switch. Therefore, we next focused on this time period. We analyzed the feeding behavior of 8-week-old C57BL/6 J male mice in detail with the feeding, drinking, and activity monitoring system (FDAMS). To observe the effect of drug administration more clearly and regularly over the observation period, INI-0602 was administered daily, with intraperitoneal injections at 0800 h.

We also tested the amounts and composition of fatty acids in HFDs offered from various companies, and we tested whether they had different effects on feeding behavior. We compared two HFDs, which had similar caloric contents and similar protein/fat/carbohydrate balances, but had different amounts of long-chain SFAs, particularly palmitic acid (C16:0) and stearic acid (C18:0), which activate microglia [12] (Table 1). We found that, compared to the HFD with elevated SFAs (HFD32), the HFD rich in SFAs (HFD60) rapidly disturbed feeding patterns and increased caloric intake during the light cycle (Additional file 2: Figure S2). Because these two diets had similar caloric contents and similar protein/fat/carbohydrate balances, we reasoned that the SFA contents must have caused the differences in the feeding pattern disturbances observed.

**Table 1:** Fat composition, % fat content, and caloric content (kcal/g) of high-fat diets (HFDs) with different amounts of saturated fatty acids (HFD32 and HFD60)

Characteristic	HFD32	HFD60
Caloric content, kcal/g	5.08	5.06
PFC^a^ (%cal)	20/57/23	18/62/20
C14:0	1.1	1.7
C14:1	0.3	0.2
C15:0	0.1	0.1
C16:0	12.6	24.4
C16:1	1.2	2.4
C17:0	0.4	0.6
C17:1	0.3	0.4
C18:0	7.5	13.8
C18:1	64.3	41.8
C18:2	10.2	12.0
C18:3	0.2	0.9
Others	1.8	1.7

^a^PFC indicates the protein/fat/carbohydrate balance in each HFD

### Disturbances in feeding patterns and activity patterns persisted after the initiation of HFD feeding in mice

**Fig. 2:**
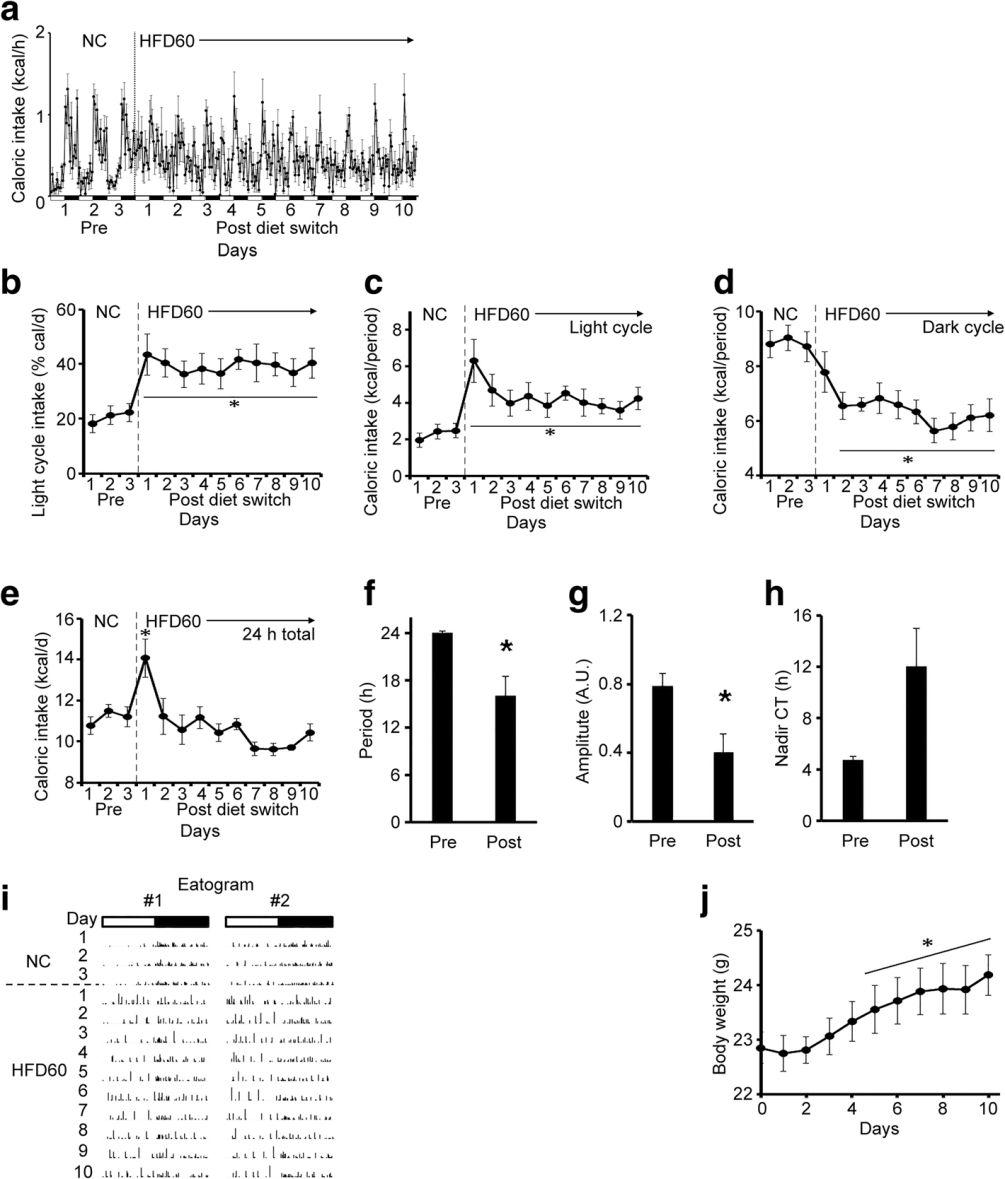
Diet switch from NC to HFD acutely affected feeding behavior in mice. Mice were acclimated to FDAMS during NC feeding, then the diet was switched from NC to HFD60 (*n* = 6) for 10 days. White and black bars on the X-axis correspond to the light and dark cycles, respectively. Vertical dashed line indicates the switch from NC (Pre) to HFD and the initiation of IP injections (Post). (**a**) Hourly caloric intake over the course of the study (1 kcal = 4.186 kJ). (**b**) The light cycle intake, expressed as the percentage of the 24-h intake. (**c**–**e**) Caloric intakes during (**c**) the light cycle, (**d**) the dark cycle, and (**e**) each 24-h period. (**f**–**h**) Cosinor analyses of feeding rhythms, including the (**f**) period length, (**g**) amplitude, and (**h**) nadir of the CT. (**i**) Eatograms of representative mice. White and black bars above the traces correspond to the light and dark cycles, respectively. (**j**) Mean body weights. Data are the means ± s.e.m. Statistical significance was determined with the Student’s paired *t*-test, evaluated at each time point, for comparisons to the day before the diet switch (Pre, in **b**–**e** and day 0 in **j**). Significant differences were determined with a Student’s paired *t*-test for comparing data taken before and after the diet switch, in **f**-**h**. *P < 0.05. Abbreviations: *NC* normal chow, *HFD* high-fat diet, *A.U.* arbitrary unit, *CT* circadian time, *FDAMS* feeding drinking, and activity monitoring system

We next asked how long the disturbance in feeding pattern persisted once HFD feeding was initiated in mice fed HFD60. We found that, during the 10-day observation period, feeding patterns remained disrupted (Fig. 2a-i). Switching the diet from NC to HFD rapidly disturbed feeding patterns, by increasing intake during the light phase (Fig. 2b-c), which resulted in increased daily food intakes (Fig. 2c). In contrast, feeding decreased during the dark cycle (Fig. 2d), which probably reflected a compensation mechanism, in response to the light phase hyperphagia. This response tended to adjust the total caloric intake over 24 h. Indeed, by the second day, the 24-h caloric intake returned to the level observed before the diet switch (Fig. 2e). To characterize the biological behavioral patterns of these mice, we performed a cosinor analysis on the recorded feeding and locomotor activity patterns [20, 21]. We found that, even under the influence of the 12-h light-dark cycle, the diet change significantly disrupted the period length and the amplitude of the feeding behavior, which indicated an aberrant feeding pattern (Fig. 2f-h). We then analyzed feeding patterns in individual mouse recordings, which we termed ‘eatograms’ (i.e., actograms of the eating behavior). We found that the frequency of feeding events and amount of intake per feeding event during the light cycle increased after the diet switch (Fig. 2i); this observation was consistent with the reduced rhythmic amplitude in the feeding pattern identified with the cosinor analyses (Fig. 2g). These changes in feeding patterns were followed by a significant increase in body weight (Fig. 2j).

In addition to feeding patterns, HFDs are known to disturb activity patterns [7, 8]. Specifically, HFD feeding was shown to reduce the absolute amount of locomotor activity during the early dark cycle and the number of spontaneous activity bouts during the light cycle. Therefore, we also analyzed the locomotor activity patterns in these mice. Switching the diet from NC to the HFD diminished the peak activity observed during the early dark cycle (Additional file 3: Fig.ure S3a) and reduced the number of light-cycle activity bouts (Additional file 3: Figure S3 h), which resulted in reduced light cycle activity (Additional file 3: Fig.ure S3b). Although the total activity over the dark cycle and over the 24-h period were not affected (Additional file 3: Figure S3c-d), the activity during the early dark cycle declined to various degrees in each mouse (Additional file 3: Figure S3 h). These changes persisted during the 10-day observation period. However, the rhythmicity of locomotor activity was not affected (Additional file 3: Figure S3e-g), because the light-dark cycle is a strong zeitgeber for locomotor activity, and these experiments were performed under a 12-h light-dark cycle. Therefore, HFD feeding disrupted the patterns of feeding and activity, and these disturbances persisted throughout the time that mice received the HFD.

### INI-0602 prevented HFD-induced feeding pattern disturbances in mice

**Fig. 3:**
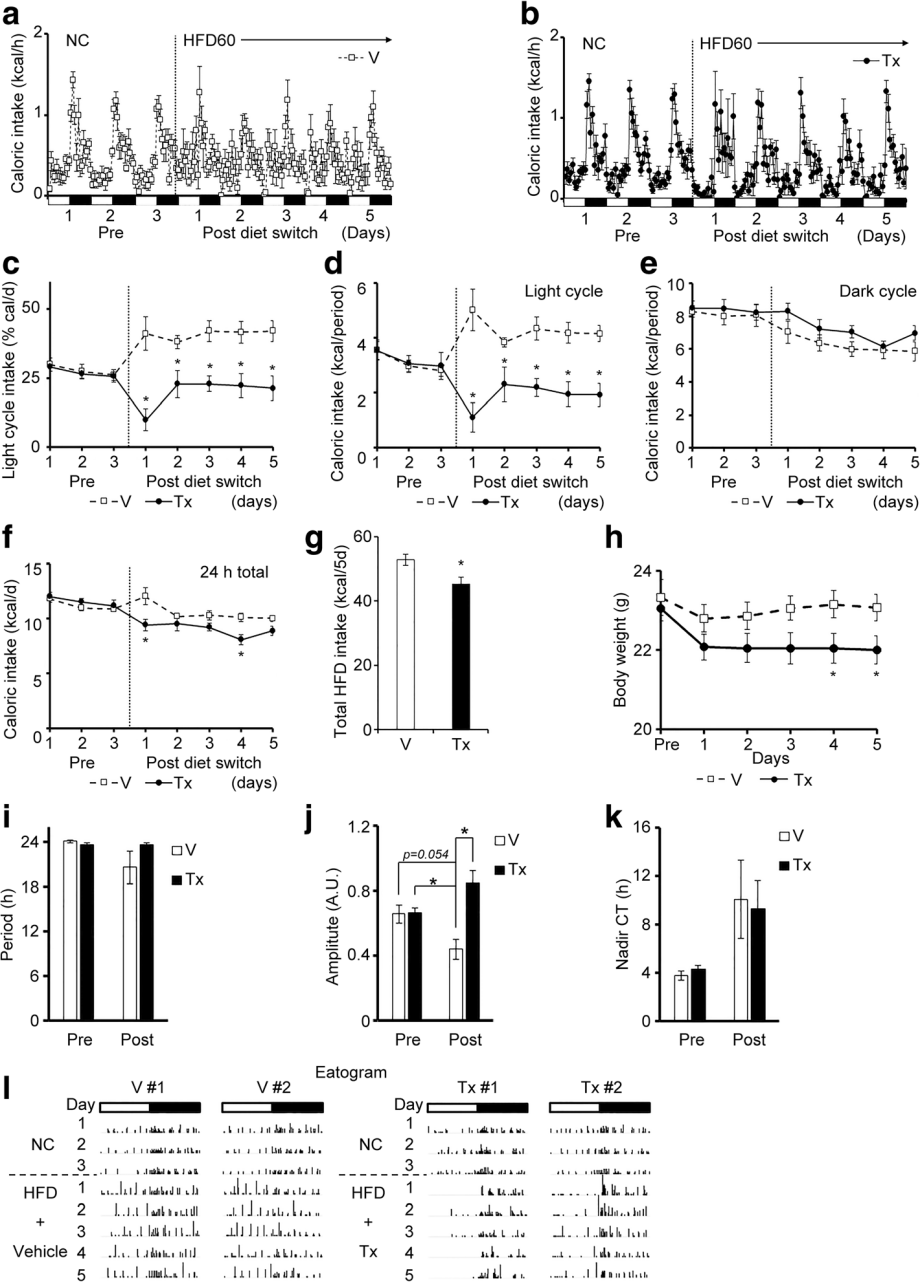
INI-0602 prevented HFD-induced feeding pattern disturbance in mice. Mice were acclimated to FDAMS with NC feeding, then switched to HFD60 with daily intraperitoneal administrations of vehicle (V, *n* = 7, white squares with dashed line) or INI-0602 (Tx, *n* = 8, black circles with solid line) for 5 days. White and black bars on the X-axis correspond to the light and dark cycles, respectively. Vertical dashed line indicates the switch from NC (Pre) to HFD and the initiation of IP injections (Post). (**a-b**) Hourly caloric intakes, measured over the course of the study (1 kcal = 4.186 kJ) in (**a**) the vehicle group and (**b**) the treatment group. (**c**) The light cycle intake, expressed as a percentage of the 24-h intake. (**d**–**g**) Caloric intakes during (**d**) the light cycle, (**e**) the dark cycle, (**f**) each 24-h period, and (**g**) over the 5-day HFD feeding period. (**h**) Mean body weights over the 5-day HFD feeding period. (**i**–**k**) Cosinor analyses of the feeding rhythms, including the (**i**) period length, (**j**) amplitude, and (**k**) nadir of the CT. (**l**) Eatograms of two representative mice in each group. White and black bars above the traces correspond to the light and dark cycles, respectively. Data are the means ± s.e.m. Statistical significance was determined with the Student’s *t*-test, for comparisons between the two groups at each time point, in **c**–**h**; Significant differences were determined with a one-way ANOVA with *post-hoc* Student’s *t*-test and the Bonferroni correction for comparisons among groups, in **i**-**k**. *P < 0.05. Abbreviations: *NC* normal chow, *HFD* high-fat diet, *A.U.* arbitrary unit, *CT* circadian time, *FDAMS* feeding drinking, and activity monitoring system

Next, we addressed the role of gap junction hemichannels in the C16:0/C18:0-rich HFD-induced feeding pattern disturbances. First, we assessed whether INI-0602 could prevent the feeding pattern disturbances induced by the HFD60 (high C16:0 and C18:0 diet). Either vehicle or INI-0602 was injected intraperitoneally at 0800 h. Then, the diet was switched from NC to HFD60. Daily intraperitoneal injections were always given at the beginning of the light cycle (0800 h). The INI-0602 treatment significantly prevented the HFD-induced increase in light cycle intake. Indeed, after initiating the HFD, we observed reductions in the caloric intake during the light-cycle, expressed as a percentage of the 24-h intake, or as the caloric intake per light cycle period. In contrast, the vehicle injection did not alter the HFD effects (Fig. 3a-d). Moreover, the INI-0602 treatment did not significantly affect the dark cycle intake (Fig. 3e). These changes in 24-h caloric intakes were only significant on days 1 and 4 (Fig. 3f). However, the total caloric intake over the 5-day HFD feeding period was significantly lower in the INI-0602–treated group than in the vehicle-treated group (Fig. 3g). These responses significantly reduced weight gain in the INI-0602 group compared to the vehicle group (Fig. 3h). The diet switch caused a significant decline in the amplitude of the feeding rhythm (i.e., the ingestion pattern showed less clear “on-off” times) in the vehicle group, but not in the INI-0602 group (Fig. 3i-k). Representative eatograms of individual mice showed that mice in the vehicle group increased the number of ingestion events and calories ingested per event during the light cycle intake; in contrast, this response was prevented in the INI-0602 group (Fig. 3l). Therefore, INI-0602 treatment prevented feeding pattern disturbances and subsequent weight gain, after switching the diet from NC to HFD in mice.

### INI-0602 did not prevent HFD-induced locomotor activity disturbances in mice

**Fig. 4:**
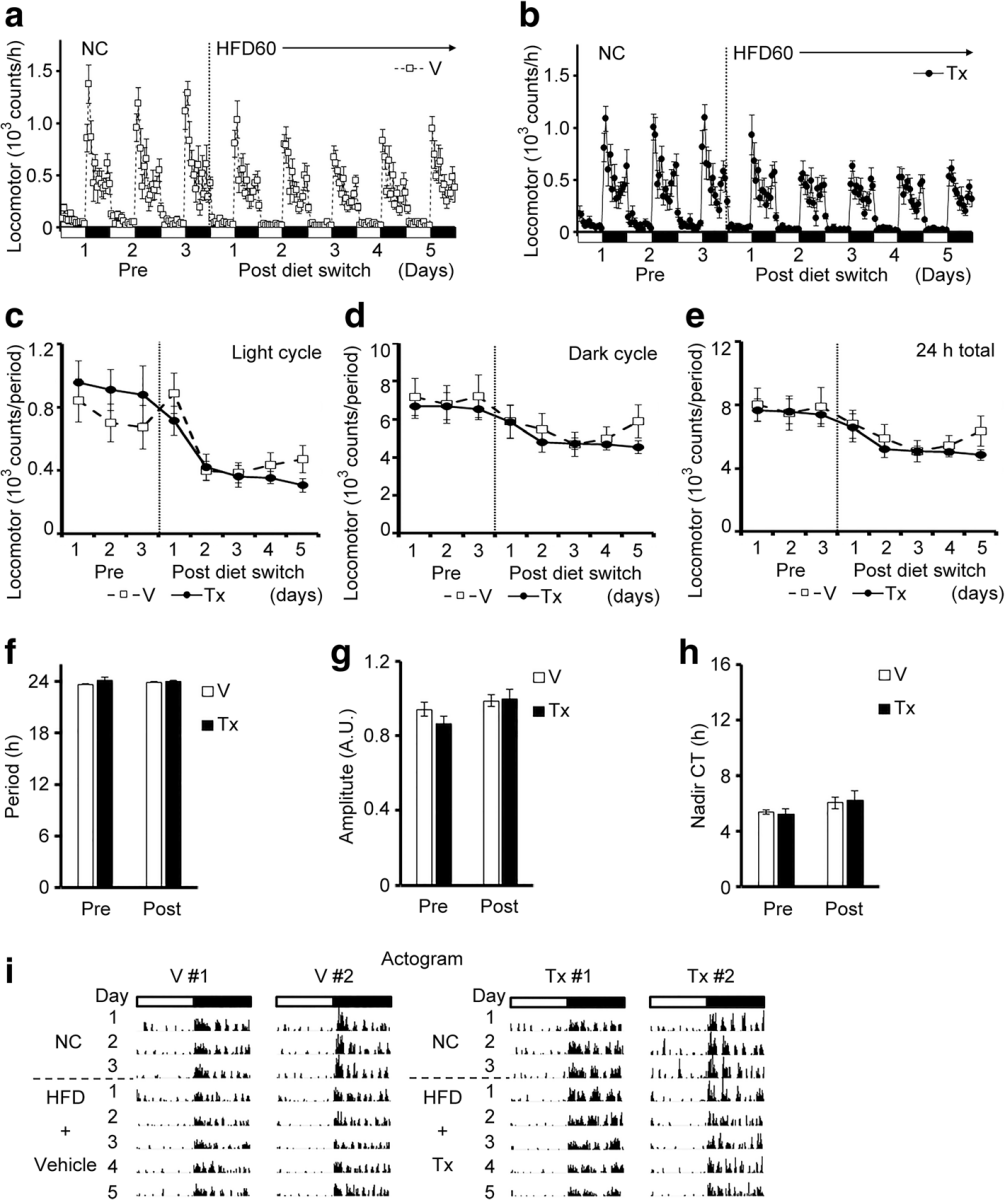
INI-0602 did not prevent HFD-induced locomotor activity pattern disturbances in mice. Locomotor activities were measured in the same mice shown in Fig. 4, for the vehicle group (V, *n* = 7, white squares with dashed line) and the treatment group (Tx, *n* = 8, black circles with solid line). White and black bars on the X-axis correspond to the light and dark cycles, respectively. Vertical dashed line indicates the switch from NC (Pre) to HFD and the initiation of IP injections (Post). (**a**–**b**) Hourly locomotor activity patterns, measured over the course of the study. Locomotor activity is shown for (**a**) vehicle (**b**) or INI-0602 groups. (**c**–**e**) Locomotor activities measured during (**c**) the light cycle, (**d**) the dark cycle, and (**e**) each 24-h period. (**f**–**h**) Cosinor analyses of the locomotor activity rhythms, including the (**f**) period length, (**g**) amplitude, and (**h**) nadir of the CT. (**i**) Actograms of two representative mice in each group. White and black bars above the traces correspond to the light and dark cycles, respectively. Data are the means ± s.e.m. Statistical significance was determined with the Student’s *t*-test, for comparisons between the two groups at each time point, in **a**–**c**; *P < 0.05. Abbreviations: *NC* normal chow, *HFD* high-fat diet, *A.U.* arbitrary unit, *CT* circadian time

Considering that INI-0602 prevented HFD-induced feeding pattern disturbances, we also assessed whether it prevented HFD-induced activity pattern disturbances in the same mice. In contrast to our expectations, INI-0602 did not prevent disturbances in locomotor activity patterns upon switching the diet from NC to HFD60 (Fig. 4a-i). The peak activities during the early dark cycle were diminished equally in both groups (Fig. 4a, b ,i). The numbers of activity bouts during the light cycle were also diminished in both groups (Fig. 4i). Thus, the rhythmicity of locomotor activities was not significantly different between the two groups (Fig. 4f-h). These results indicated that INI-0602 had differential effects on the feeding and activity patterns disturbances induced by HFDs.

### INI-0602 did not affect feeding or the activity pattern of male mice fed the NC diet

**Fig. 5:**
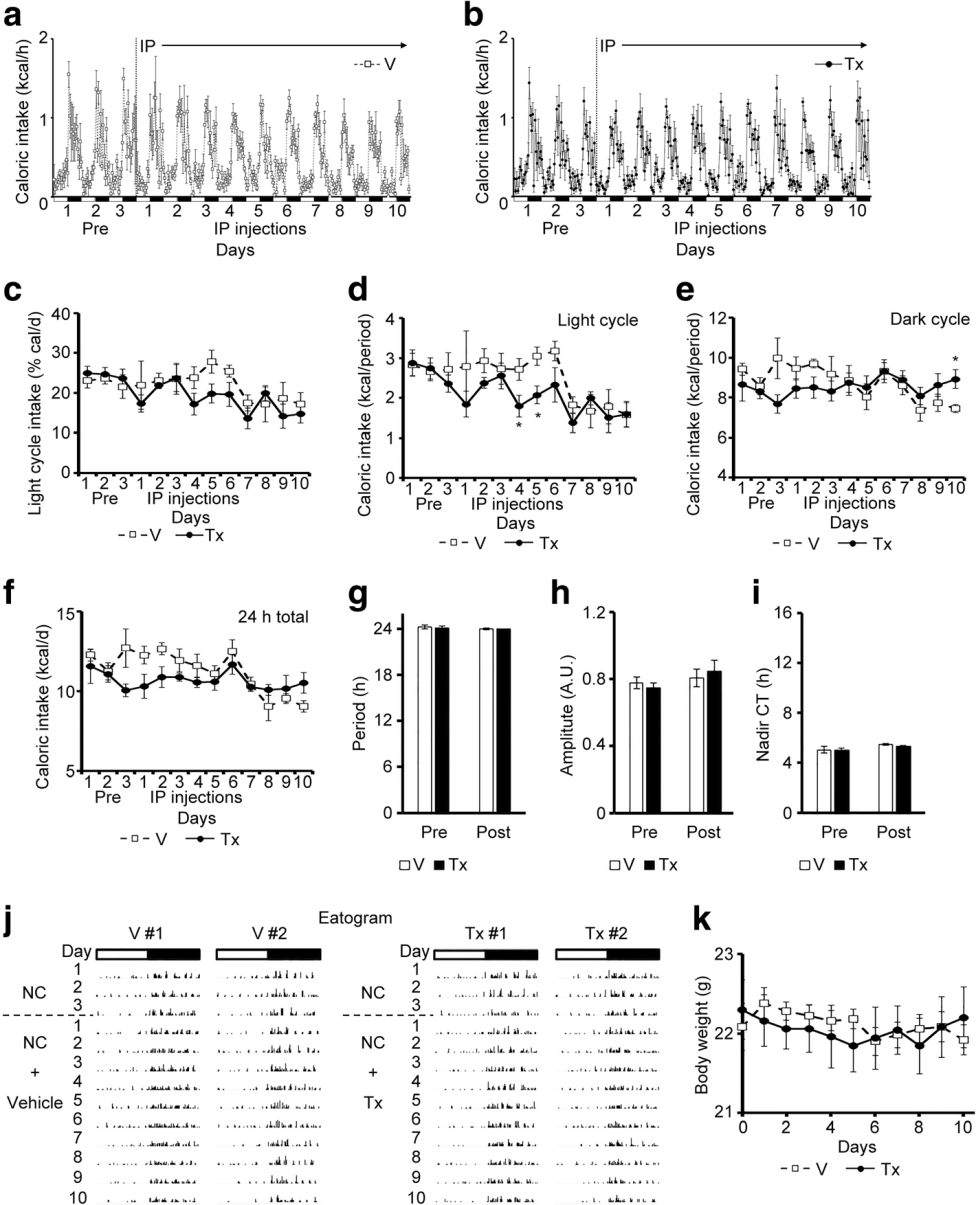
INI-0602 did not affect feeding behavior or body weight in mice fed normal chow (NC). Mice were acclimated to FDAMS with NC feeding, then received intraperitoneal administration of vehicle (V, *n* = 5, white squares with dashed line) or INI-0602 (Tx, *n* = 5, black circles with solid line) every day for 10 days. White and black bars on the X-axis correspond to the light and dark cycles, respectively. Vertical dashed lines indicate the switch between no injection (Pre) and the initiation of IP injections (Post). (**a-b**) Hourly caloric intakes over the course of the study (1 kcal = 4.186 kJ) are shown for (**a**) the vehicle group and (**b**) the treatment group. (**c**) The light cycle intake, expressed as a percentage of the 24-h intake. (**d**–**f**) Mean daily caloric intakes during (**d**) the light cycle, (**e**) the dark cycle, and (**f**) each 24-h period. (**g**–**i**) Cosinor analyses of the feeding rhythms, including the (**g**) period length, (**h**) amplitude, (**i**) and nadir of the CT. (**j**) Eatograms of two representative mice in each group. White and black bars above the traces correspond to the light and dark cycles, respectively. (**k**) Mean body weights. Data are the means ± s.e.m. Statistical significance was determined with the Student’s *t*-test, for comparisons between the two groups at each time point, in **b**–**e** and **k**. Significant differences were determined with a one-way ANOVA with the *post-hoc* Student’s *t*-test and the Bonferroni correction for comparisons among groups, in **g-i**. *P < 0.05. Abbreviations: *NC* normal chow, *A.U.* arbitrary unit, *CT* circadian time, *IP* intraperitoneal, *FDAMS* feeding drinking, and activity monitoring system

We also analyzed the effect of INI-0602 on feeding behavior, locomotor activity, and body weight in NC-fed mice. We observed no differences in the feeding pattern, locomotor activity, or body weight between mice that received INI-0602 injections and those that received vehicle injections (Fig. 5 and Additional file 4: Figure S4). Therefore, INI-0602 did not affect the feeding pattern, locomotor activity, or body weight in NC-fed mice.

### INI-0602 did not attenuate hypothalamic microglial activation or inflammatory cytokine induction in mice

It is known that HFD feeding acutely activates microglia, specifically in the ARC of the hypothalamus [11]. The ARC integrates humoral information from the periphery and regulates feeding and body weight [15]. After a diet switch from NC to HFD, acute hypothalamic inflammation occurs within the first 3 days, which is characterized by microglial activation, followed by secondary astrocytic inflammation in 7 days [11]. Because the HFD-induced feeding pattern disturbance and the preventive effect of INI-0602 both occurred within the first 3 days, we assessed the effects of INI-0602 on ARC microglial activation, after 1 or 3 days of HFD60 feeding. Immunohistochemistry analyses of Iba-1 showed that neither the Iba-1(+) cell number nor the Iba-1(+) area within the ARC was affected by INI-0602 (Additional file 5: Figure S5a-f). These results indicated that INI-0602 did not block HFD-induced acute microglia activation. Therefore, the data suggested that INI-0602 acted downstream of HFD-induced microglial activation to prevent feeding pattern disturbances (Additional file 5: Figure S5 h).

It is known that activated microglia evoke neuroinflammation through two pathways: gap junction hemichannels and inflammatory cytokine expression [17]. Therefore, at 1 and 3 days after switching to HFD60, we measured hypothalamic expression levels of the microglia-specific marker, *Tmem119* [22], and genes in the inflammatory cytokine pathway (*Ikbkb* and *Tnfa*). We found that the HFD60-induced changes in the expression of these genes were not significantly affected by INI-0602 (Additional file 5: Figure S5 g). Taken together, these findings suggested that INI-0602 prevented HFD-induced feeding pattern disturbances without significantly affecting microglia activation (the upstream event) or the induction of inflammatory cytokines (another pathway). Although we lack the direct evidence, our results implied that an INI-0602 block of the gap junction hemichannel pathway might be one mechanism involved in preventing HFD-induced feeding pattern disturbances (Additional file 5: Figure S5 h).

### INI-0602 given after the switch to HFD feeding failed to restore existing HFD-induced feeding and activity disturbances

**Fig. 6:**
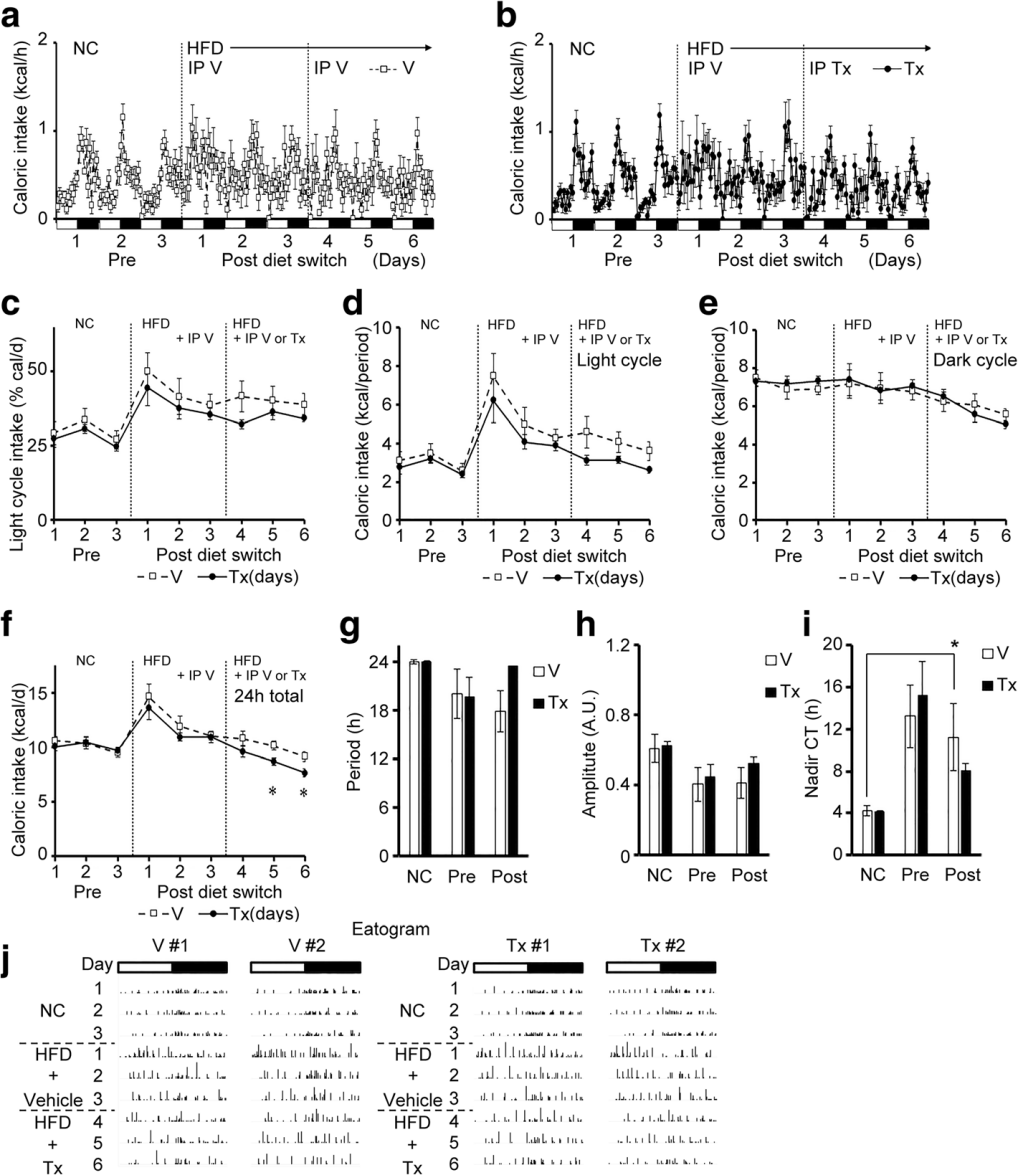
INI-0602 given after HFD feeding did not restore the normal feeding pattern in mice. (**a**–**f**) Mice were acclimated to FDAMS with NC feeding, then switched to HFD60 with intraperitoneal administration (IP) of vehicle for 3 days, followed by injections of vehicle (V, *n* = 6, white squares with dashed line) or INI-0602 (Tx, *n* = 6, black circles with solid line) every day for 3 days. White and black bars on the X-axis correspond to the light and dark cycles, respectively. Two vertical dashed lines indicate (*first line*) the switch from NC (Pre) to HFD and the initiation of vehicle IP injections, and then (*second line*) the switch (Post) to either vehicle or INI-0602 (Tx) injections. (**a-b**) Hourly caloric intakes over the course of the study (1 kcal = 4.186 kJ) are shown for (**a**) the vehicle group and (**b**) the treatment group. (**c**) The light cycle intake expressed as a percentage of the 24-h intake. (**d**–**f**) Caloric intakes during (**d**) the light cycle, (**e**) the dark cycle, and (**f**) each 24-h period. (**g**–**i**) Cosinor analyses of the feeding rhythms, including the (**g**) period length, (**h**) amplitude, and (**i**) nadir of the CT. (**j**) Eatograms of two representative mice in each group. White and black bars above the traces correspond to the light and dark cycles, respectively. Data are the means ± s.e.m. Statistical significance was determined with the Student’s *t*-test, for comparisons between the two groups at each time point, in **c**–**f**. Significant differences were determined with a one-way ANOVA with a *post-hoc* Student’s *t*-test and the Bonferroni correction for comparisons among groups, in **g**-**i**. *P < 0.05. Abbreviations: *NC* normal chow, *HFD* high-fat diet, *A.U.* arbitrary unit, *CT* circadian time, *FDAMS* feeding drinking, and activity monitoring system

We next tested whether INI-0602 could reverse the HFD-induced disturbances in feeding and activity patterns. Mice were fed HFD for 3 days to disturb feeding and activity patterns. Then, INI-0602 injections were initiated. Three-day INI-0602 treatments did not alter the existing feeding rhythm disturbances induced by HFD (Fig. 6a-j); the percentage of light cycle intake remained elevated compared to the pre-diet percentages (Fig. 6c). Interestingly, on the second and third days of INI-0602 treatment (Days 5 and 6 of the experiment), daily caloric intake was significantly reduced compared to vehicle-treated mice (Fig. 6f). However, INI-0602 treatment did not reverse HFD-induced activity pattern disturbances (Additional file 6: Figure S6a-i). Therefore, once HFD caused disturbances in feeding and activity, INI-0602 could not restore the behaviors to normal.

## Discussion

In this work, we used a central-acting reagent, INI-0602, which blocks the gap junction hemichannel pathway, but not the inflammatory cytokine pathway, during neuroinflammation. We analyzed its effects on feeding and locomotor activity patterns in detail, within the context of HFD-induced obesity. We addressed two major questions; first, how does HFD disturb feeding and locomotor activity patterns at the behavioral levels? Second, does the gap junction hemichannel pathway play any role in the pathogenesis of HFD-induced behavioral pattern disturbances and obesity (independently of the inflammatory cytokine signaling pathway). We found that HFD caused acute hyperphagia mainly by increasing light cycle feeding; moreover, the feeding pattern disturbances worsened with increasing proportions of long-chain SFAs in the HFD. These results suggested that the long-chain SFAs in HFDs were responsible for disturbing feeding rhythms. We found that INI-0602 prevented HFD-induced obesity and HFD-induced feeding pattern disturbances, but not HFD-induced activity pattern disturbances. However, INI-0602 failed to restore an existing HFD-induced feeding pattern disturbance, when it was given after HFD feeding had been initiated. These results suggested that the gap junction hemichannel pathway mediated the effect of HFD feeding on the feeding pattern only at the beginning phase of the disturbance. Importantly, preventing the initial step was sufficient to attenuate the degree of HFD-induced obesity in mice, despite HFD-induced locomotor activity pattern disturbances.

Long-chain SFAs are known to activate microglia within the ARC and induce inflammatory cytokines [12]. The initial hypothalamic inflammation is associated with the acute activation of microglia within 3 days followed by the activation of astrocytes in 7 days [11]. HFD-responsive microgliosis occurs specifically in the ARC and not in the adjacent ventromedial nucleus of the hypothalamus [11, 23]. The activation of the ARC residential microglia induces the subsequent recruitment of peripheral myeloid cells to the ARC by 4 weeks of HFD ingestion, further promoting ARC microgliosis [23]. These microglial inflammation further triggers astrocyte inflammation, which also contribute to the establishment of the diet-induced obesity [24]. Therefore, the NF-κB-dependent microglial activation is critical for the entry of bone-marrow-derived myeloid cells to the ARC, propagation of the hypothalamic inflammation, and the subsequent development of the HFD-induced obesity. The instant disruption of feeding rhythm upon HFD ingestion suggested the involvement of the acute hypothalamic ARC inflammation, which is mostly mediated by microglia.

In addition to the induction of the NF-κB-dependent inflammatory cytokine signaling, neuroinflammation activates the gap junction hemichannel pathway [17]. This hemichannel pathway is blocked by INI-0602, without affecting the acute induction of inflammatory cytokines. Here, we demonstrated that the INI-0602 block prevented HFD-induced feeding pattern disturbances. Therefore, we speculated that HFD-induced obesity had two components; the gap junction hemichannel pathway, which affected “when you eat”, and the inflammatory cytokine signaling pathway, which affected “how much you eat.”

Gap junctions consist of two apposed hemichannels, each contributed by one cell. They are used for intercellular diffusion of second messengers smaller than 1 kDa, such as Ca^2+^, IP3, and nucleotides [25]. Under physiological conditions, astrocytic gap junctions contribute to the stability of neuronal networks; in contrast, resting microglia express low to undetectable levels of connexins (components of gap junction hemichannels). However, under inflammatory conditions, astrocytic gap junctions are shut down by classical inflammatory mediators, and the expression of connexin is induced in activated microglia, and morphological changes induced in microglia upon activation result in the detachment of gap junctions and cell adhesions and form unapposed hemichannels [25, 26]. Through these unapposed hemichannels, activated microglia release massive amounts of pro-inflammatory factors, such as ATP and glutamate, into the extracellular space. This milieu causes neuronal damage and secondary glial inflammation [17, 26]. Our findings suggested that SFAs activated this gap junction hemichannel pathway and led to feeding pattern disruptions.

These observations have raised the question: what are the identities of the small molecules that mediate HFD-induced feeding rhythm disturbances? Within the context of neurodegenerative disease models, in which extracellular glutamate plays pivotal roles, we previously showed that INI-0602 prevented lipopolysaccharide-induced microglial glutamate release in vivo and in vitro, and prevented disease progression [18]. However, the identities of the pathogenic small molecules involved in HFD-induced feeding pattern disturbances remain unknown, because doing microdialysis in the ARC is challenging even in rats and no previous report exists in mice. Furthermore, the microdialysis of the ARC in HFD-fed rat has not been reported. Therefore, in this study, we could not directly determine whether INI-0602 blocked the release of small molecules in the hypothalamus that were relevant to HFD-induced feeding disturbances. This issue must be addressed in future studies.

Interestingly, INI-0602 had differential effects on HFD-induced disturbances in feeding and locomotor activity. The suprachiasmatic nucleus (SCN) of the hypothalamus serves as the master clock; this clock systemically coordinates various biological rhythms by regulating slave clocks located within the brain (such as “feeding clock”, “activity clock”, etc.) and the periphery. This control is demonstrated in the rhythmic expression patterns of clock genes, such as *period1* [27–29]. Both feeding and ambient light serve as zeitgebers (timing cues from the external environmental). It is generally believed that HFDs disturb clocks in the gastrointestinal tract (including the liver), and feeding zeitgebers indirectly reset the phase of the SCN clock through a feedback loop that includes humoral and neural pathways. However, our finding that INI-0602 had differential effects on HFD-induced rhythm disturbances suggested that INI-0602 did not act on the master clock in the SCN to rescue HFD-induced behavioral disturbances. Moreover, a previous study showed that a 12-week HFD feeding period had no effect on the molecular oscillations in the SCN [30]. Therefore, it is unlikely that INI-0602 restored the rhythmicity of the feeding clock by blocking only the specific influence of the SCN rhythm on the feeding clock (and not its influence on the activity clock). Instead, we hypothesized that the HFD might have disturbed both the feeding clock and the activity clock through two independent mechanisms; thus, INI-0602 treatment could have blocked only the former and not the latter. However, the precise location of the feeding clock in the brain remains an issue of debate. Proposed candidates include the ARC [31, 32], NPY neurons [33, 34], and the dopamine system [35], among others. Moreover, INI-0602 action is not specific for any particular location in the CNS. Therefore, the identity of the feeding clock remains to be uncovered in future investigations.

The findings of this study have shed some light to how the intrinsic feeding rhythm is disturbed by HFDs, and how these disturbances might be prevented. In mice fed ad libitum, the feeding pattern is presumably controlled by the intrinsic feeding drive. In humans, the timing of meals is determined by an intrinsic feeding drive (hunger) combined with the social environment and other factors [1]. A disruption in a meal schedule (e.g., breakfast, lunch, dinner) could induce hunger-related stress and lead to in-between-meal snacking. Snacks are often rich in fat, which would exacerbate the disturbance in the feeding rhythm. Therefore, we propose that normalizing the intrinsic rhythm of the feeding drive would be beneficial in humans by facilitating a regular feeding schedule and reducing snacking. Identification of the pathogenic small molecule(s) responsible for HFD-induced feeding rhythm disturbances would help improve our strategies for improving the feeding pattern to promote better health.

## Methods

### Animals, diets, and housing conditions

All experimental procedures were performed in accordance with the Guide for the Care and Use of Laboratory Animals of the Science Council of Japan, and they were approved by the Animal Experiment Committee of Gunma University. All experiments were performed in wild-type C57BL/6 J male mice, obtained from CLEA Japan (Tokyo, Japan). Male mice, ages 7–11 weeks, were maintained on a 12-h light/dark cycle at 25 °C and they were given free access to NC and tap water before the experiment. The NC (CE-2) and HFD (HFD32) were purchased from CLEA Japan. Another HFD (HFD60) was purchased from Oriental Yeast Co. (Tokyo, Japan). The HFD32 and HFD60 had different fatty acid compositions (Table 1**;** information provided by manufacturers). The HFD portions were changed daily to prevent oxidation of fatty acids in the diet. The light/dark cycle was 0600 h/1800 h in the 4-week study and 0800 h/2000 h in the studies that employed the feeding, drinking, and activity monitoring system (FDAMS).

### Food intake, feeding behavior, and locomotor activity measurements

In the 4-week study, a multifeeder purchased from Shinfactory (Fukuoka, Japan) was used to measure food intake. Food intake was measured daily at 0800 h. In the experiments that employed FDAMS (also purchased from Shinfactory), food intake and locomotor activity were recorded every minute and analyzed over a specified period with Excel, as described previously [36]. The FDAMS monitored food intake, food access, locomotor activity, and water consumption every minute. Mice had to stick their heads into the food port to gain access to the food. This apparatus prevented mice from taking away large chunks of food, because mice could not use their paws to grasp food. We also prevented the measurement of shredded food as food intake by placing the food container below the mouse’s body; thus, all shredded food remained in the food container. Caloric intakes (1 kcal = 4.186 kJ) were calculated based on the grams of diet consumed (NC, 3.449 kcal/g; HFD32, 5.076 kcal/g; HFD60, 5.062 kcal/g).

### Eatogram and actogram analyses

Feeding and activity data were analyzed in 10-min bins to generate eatograms of eating behavior [37] and actograms of locomotor activity. Caloric intake was plotted in 0.1 kcal/10 min bins, and the height of each bar corresponded to the amount of calories ingested. Locomotor activity was plotted in 100 count/10 min bins, and the height of each bar indicated the amount of activity.

### Cosinor rhythmometric analyses of feeding and locomotor activities

We analyzed the rhythms of hourly feeding behavior and locomotor activity with the cosinor-rhythmometry method [20, 21] and analysis software (Kai-Seki Ninja SL00–01; Churitsu Electric Corporation, Nagoya, Aichi, Japan). The amplitude of each rhythm was calculated as the average peak-to-trough ratio of all the cycles. The period length represented the length of one rhythmic cycle. We also quantified circadian time (CT), which is the rhythm set by an organism’s endogenous circadian clock. The CT0 was the beginning of the subjective light cycle, and the CT12 was the beginning of the subjective dark cycle. The CT nadir represented the nadir phase of the rhythmic cycle, adjusted to a 24-h rhythmic cycle.

### Preparation of INI-0602

INI-0602 is a carbenoxolone derivative. We produced it in-house, in batch quantity, as previously described [18]. It can also be purchased at Wako Pure Chemical Industries (Osaka, Japan). INI-0602 was first dissolved in ethanol, then mixed with saline to obtain a 5% ethanol/95% saline emulsion. We injected the emulsion (10 μl/g body weight [BW], for a dose of 20 mg/kg BW) or the vehicle (10 μl/g BW of 5% ethanol/95% saline) intraperitoneally into mice with a 30 G needle. Injections were performed at 0800.

### INI-0602 administration protocol

The dosing of INI-0602 was based on a previous study that investigated the efficacy and safety of INI-0602 administration in murine models of neurodegenerative diseases [18]. Intravenous injections of INI-0602 at 20 mg/kg BW in mice caused rapid accumulation of INI-0602 in brain, but the plasma concentration of the drug rapidly declined over the same time-frame; this resulted in high brain-to-plasma drug concentration [18]. Previous studies showed that intraperitoneal injections of INI-0602 at 20 mg/kg BW every other day could effectively suppress disease progression in neurodegenerative mouse models. Moreover, it did not cause side effects in peripheral tissues, even after chronic administration for 5 months [18]. In the present, 4-week HFD-feeding study, we used the same administration protocol. For other short-term experiments, we administered INI-0602 intraperitoneally to mice every day, at the beginning of the light cycle.

### Body weight and body composition, measured with computed tomography

Body weight was measured at 0800 h every day. Body composition was measured with a computed tomography scan at the end of the 4-week study. Briefly, mice were anesthetized, and computed tomography imaging was performed with a LaTheta in vivo Micro computed tomography scanner, purchased from Hitachi (Tokyo, Japan). Adiposity was measured as previously described with the software provided with the computed tomography scanner [38].

### Immunohistological analyses of the ARC of the hypothalamus

Mice were anesthetized with pentobarbital, and euthanized between 0800 h and 1000 h. Next, mice were perfused with phosphate-buffered saline, followed by 4% paraformaldehyde. The brain samples were fixed overnight, and fixed-frozen sections of hypothalamic ARC were used for immunohistological analyses of microglia, as described previously [39]. Immunohistochemical staining was performed with the anti-Iba1 rabbit antibody (Wako Pure Chemical Industries, 019–19,741, 1:1000 dilution), as primary antibody, and the Cy3-conjugated AffiniPure donkey anti-rabbit IgG (H + L) (Jackson ImmunoResearch, 711–165-152, 1:200 dilution) as secondary antibody. Images were acquired with a fluorescent microscope, BZ-9000 (Keyence, Osaka, Japan). The Iba-1(+) signals were measured with the densitometry function in ImageJ software, as described previously [40]. We analyzed four ARC sections per animal.

### RNA isolation and quantitative RT-PCR

RNA isolation and cDNA synthesis were performed as previously described [39], with RNAiso plus (TAKARA BIO, Otsu, Japan) and the Improm II reverse transcription system (Promega, Tokyo, Japan). Real-time PCR was performed with the LightCycler system and LightCycler 480 SYBR Green I (Roche Diagnostics K.K., Tokyo, Japan). The following primer pairs were used: *Actb*, forward: 5’-AGCCTTCCTTCTTGGGTA-3′ and reverse: 5’-GAGCAATGATCTTGATCTTC-3′; *Ikbkb*, forward: 5’-CTGAAGATCGCCTGTAGCAAA-3′ and reverse: 5’-TCCATCTGTAACCAGCTCCAG-3′; *Tmem119*, forward: 5’-CACCCAGAGCTGGTTCCATA-3′ and reverse: 5’-GTGACACAGAGTAGGCCACC-3′; and *Tnfa*, forward: 5’-ACGGCATGGATCTCAAAGAC-3′ and reverse: 5’-AGATAGCAAATCGGCTGACG-3′.

### Statistical analysis

All values are presented as the mean ± s.e.m. Differences between groups and differences from the beginning time point were analyzed with the Student’s *t*-test. For cosinor rhythmometric analyses, we performed one-way analyses of variance (ANOVA) and a post-hoc Student’s *t*-test with Bonferroni’s correction. Results with a *P* < 0.05 were considered statistically significant.

## Additional files

Additional file 1: **Figure S1.** INI-0602 did not affect microglia in the ARC after 4 weeks of HFD feeding in mice. (**a**-**c**) Histological analyses of tissue sections of the ARC of the hypothalamus, dissected from mice that underwent the 4-week study. (**a**) Representative photomicrographs of ARC sections show microglia stained with anti-Iba-1 (*red*) and nuclei stained with DAPI (*blue*). Quantifications show (**b**) Iba-1 (+) cell numbers (**c**) and Iba-1 (+) areas. Four coronal sections were analyzed per mouse. # inidicates the section number, from rostral to caudal. Data are the means ± s.e.m. Statistical significance was determined rostral to caudal. Data are the means ± s.e.m. Statistical significance was determined with the Student’s *t*-test, for comparisons between the two groups at each time point. *P<0.05. Abbreviations: A.U., arbitrary unit; eWAT, epididymal white adipose tissue; HFD, high-fat diet; wt, weight. (TIF 1021 kb)

Additional file 2: **Figure S2.** The SFAs in HFDs disrupted feeding patterns by increasing light cycle intake. (**a**–**c**) Mice were fed one of two diets high in saturated fatty acids (SFAs); the HFD60 contained higher amounts of C16:0 and C18:0 (*n* = 6, black circles with solid line) than the HFD32 (*n* = 8, white squares with dashed line). After acclimation to FDAMS with NC, mice were fed the HFDs for 5 days. White and black bars on the X-axis correspond to the light and dark cycles, respectively. (**a**-**b**) Hourly caloric intake (1 kcal = 4.186 kJ) before (Pre) and after the diet switch from NC to (**a**) HFD60 or (**b**) HFD32. (**c**) The light cycle intake expressed as a percentage of the 24-h intake. Black circles with solid line: the HFD60 group; white squares with dashed line: the HFD32 group. Data are the means ± s.e.m. Statistical significance was determined with the Student’s paired *t*-test for comparisons to pre-diet values for each group, in c; *P < 0.05. Abbreviations: NC, normal chow; HFD, high-fat diet; FDAMS, feeding drinking, and activity monitoring system. (TIF 226 kb)

Additional file 3: **Figure S3.** Diet switch from NC to HFD acutely affected locomotor activity patterns in mice. Locomotor activity data for the same mice that were analyzed in Fig. 2. White and black bars on the X-axis correspond to the light and dark cycles, respectively. Vertical dashed line indicates the switch from NC (Pre) to HFD and the initiation of IP injections (Post). (**a**) Hourly locomotor activity over the course of the study. (**b**–**d**) Locomotor activity during (**b**) the light cycle, (**c**) the dark cycle, and (**d**) each 24-h period. (**e**-**g**) Cosinor analyses of the locomotor activity rhythms, including the (**e**) period length, (**f**) amplitude, and (**g**) nadir of the CT. (**h**) Actograms of two representative mice in each group. White and black bars above the traces correspond to the light and dark cycles, respectively. Data are the means ± s.e.m. Statistical significance was determined with the Student’s paired *t*-test, evaluated at each time point, for comparisons to the day before the diet switch (Pre in **b**–**d**, and day 0 in **h**). Significant differences were determined with a Student’s paired t-test for comparing data taken before and after the diet switch, in **e**-**g**. *P < 0.05. Abbreviations: A.U., arbitrary unit; CT, circadian time. (TIF 737 kb)

Additional file 4: **Figure S4.** INI-0602 did not affect locomotor activity in mice fed a normal chow diet. Locomotor activity data for the same mice that were analyzed in Fig. 5, treated with vehicle (V, *n* = 5, white squares with dashed line) or INI-0602 (Tx, *n* = 5, black circles with solid line). White and black bars on the X-axis correspond to the light and dark cycles, respectively. (**a**–**b**) Hourly locomotor activity pattern is shown before (Pre) and after (Post) initiation of intraperitoneal (IP) injections of (**a**) vehicle or (**b**) INI-0602. (**c**–**e**) Locomotor activity during (**c**) the light cycle, (**d**) the dark cycle, and (**e**) each 24-h period. (**f**–**h**) Cosinor analyses of the locomotor activity rhythms, including the (**f**) period length, (**g**) amplitude, and (**h**) nadir of the CT. (**i**) Actograms of two representative mice in each group. White and black bars above the traces correspond to the light and dark cycles, respectively. Data are the means ± s.e.m. Statistical significance was determined with the Student’s *t*-test for comparisons between the two groups at each time point, in **c**–**e**. Significant differences were evaluated with the one-way ANOVA with a *post-hoc* Student’s *t*-test and the Bonferroni correction for comparisons among groups, in **f**-**h**. *P < 0.05. Abbreviations: A.U., arbitrary unit; CT, circadian time; NC: normal chow. (TIF 1139 kb)

Additional file 5: **Figure S5.** INI-0602 did not block HFD-induced microglial activation or inflammatory cytokine expression in the hypothalamus. Mice were fed HFD for 1 or 3 days, as indicated, and they received daily intraperitoneal injections of vehicle (V, *n* = 3-4, white symbols) or INI-0602 (Tx, *n* = 4, black symbols). (**a**-**f**) Histological analyses tissue sections of the ARC of the hypothalamus. (**a** and **d**) Representative photomicrographs of ARC sections show microglia stained with anti-Iba-1 (*red*) and nuclei stained with DAPI (*blue*). Quantifications show (b and e) Iba-1 (+) cell numbers, and (**c** and **f**) Iba-1 (+) areas. Four coronal sections were analyzed per mouse. # inidicates the section number, from rostral to caudal. (**g**) Quantitative PCR results show hypothalamic gene expression levels measured in another cohort of mice that underwent the same treatments. (**h**) The proposed point of action of INI-0602 (*red*) in the context of HFD feeding. Data are the means ± s.e.m. Statistical significance was determined with the Student’s *t*-test for comparisons between groups at each time point; *P < 0.05. Abbreviations: HFD, high-fat diet; A.U., arbitrary units. (TIF 5372 kb)

Additional file 6: **Figure S6.** INI-0602 given after initiating HFD feeding did not restore locomotor activity patterns in mice. Locomotor activity results for the same mice that were analyzed in Fig. 6, treated with vehicle (V, *n* = 6, white squares with dashed line) or INI-0602 (Tx, *n* = 6, black circles with solid line). White and black bars on the X-axis correspond to the light and dark cycles, respectively. Vertical dashed line indicates the switch from NC (Pre) to HFD and the initiation of IP injections (Post). (**a**–**b**) Hourly locomotor activity pattern over the course of the study in (**a**) the vehicle group and (**b**) the treated group. (**c**–**e**) Locomotor activity during (**c**) the light cycle, (**d**) the dark cycle, and (**e**) each 24-h period. (**f**–**h**) Cosinor analyses of the locomotor activity rhythms, including the (**f**) period length, (**g**) amplitude, and (**h**) nadir of the CT. (**i**) Actograms of two representative mice in each group. White and black bars above the traces correspond to the light and dark cycles, respectively. Data are the means ± s.e.m. Statistical significance was determined with the Student’s *t*-test for comparisons between groups at each time point, in **c**–**e**. Significant differences were determined with a one-way ANOVA with *post-hoc* Student’s *t*-test and the Bonferroni correction for comparisons among groups, in **f**-**h**. Abbreviations: HFD, high-fat diet; A.U., arbitrary unit; CT, circadian time. (TIF 1284 kb)

## Abbreviations

A.U.: arbitrary unit
ANOVA: analyses of variance
ARC: arcuate nucleus
CNS: central nervous system
CT: circadian time
eWAT: epididymal white adipose tissue
FDAMS: feeding drinking, and activity monitoring system
HFD: high-fat diet
IL-1β: interleukin-1 beta
IP: intraperitoneal
IP3: inositol triphosphate
NC: normal chow
NF-κB: nuclear factor-kappa B
SCN: suprachiasmatic nucleus
SFA: saturated fatty acid
TNF-α: tumor necrosis factor-alpha
Tx: treatment
V: vehicle
