# Supplementary figures and images for: A central-acting connexin inhibitor, INI-0602, prevents high-fat diet-induced feeding pattern disturbances and obesity in mice
Source: Mol Brain. 2018 May 24;11:28. doi: 10.1186/s13041-018-0372-9 (PMC5968494; doi:10.1186/s13041-018-0372-9)

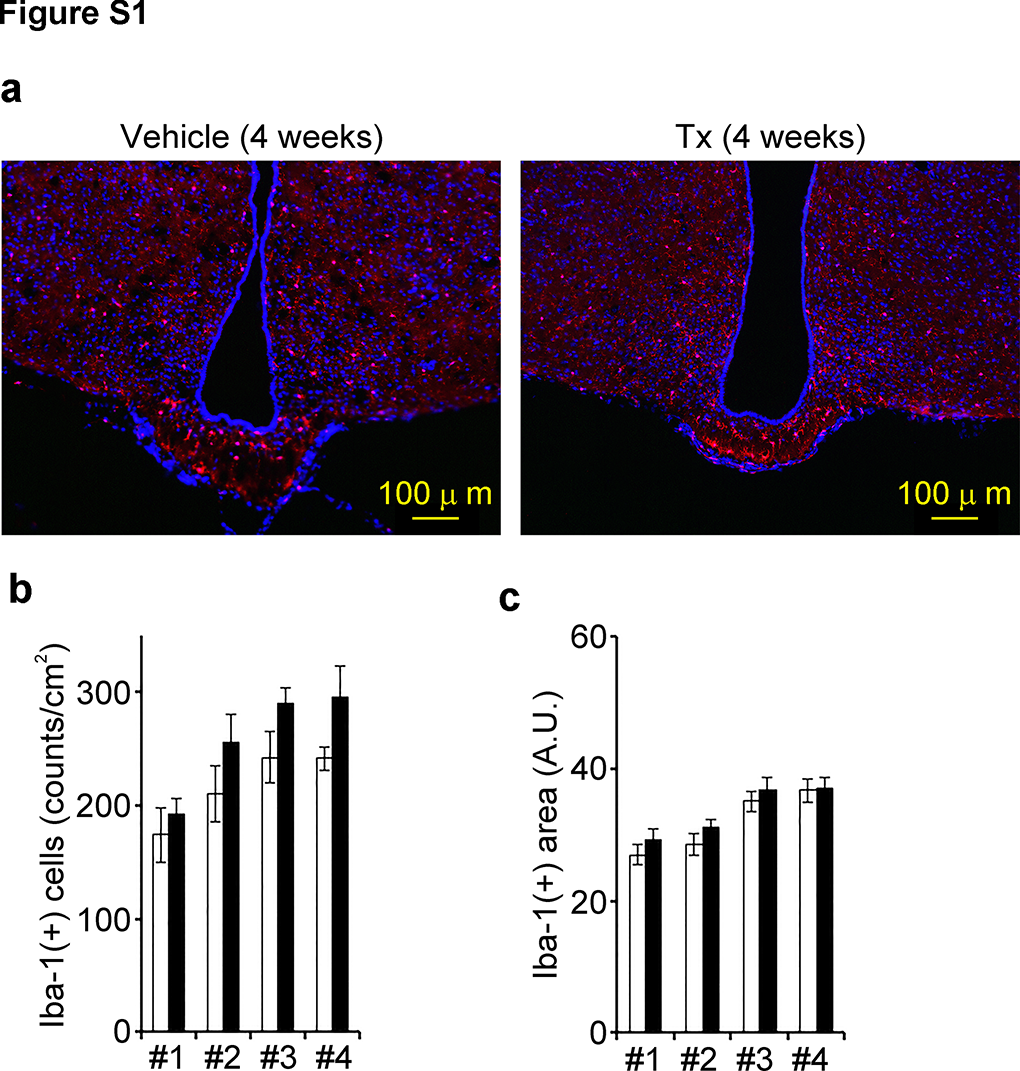

Supplement: Supplementary file 1 — Figure S1. INI-0602 did not affect microglia in the ARC after 4 weeks of HFD feeding in mice. (a-c) Histological analyses of tissue sections of the ARC of the hypothalamus, dissected from mice that underwent the 4-week study. (a) Representative photomicrographs of ARC sections show microglia stained with anti-Iba-1 (red) and nuclei stained with DAPI (blue). Quantifications show (b) Iba-1 (+) cell numbers (c) and Iba-1 (+) areas. Four coronal sections were analyzed per mouse. # inidicates the section number, from rostral to caudal. Data are the means ± s.e.m. Statistical significance was determined rostral to caudal. Data are the means ± s.e.m. Statistical significance was determined with the Student’s t-test, for comparisons between the two groups at each time point. *P<0.05. Abbreviations: A.U., arbitrary unit; eWAT, epididymal white adipose tissue; HFD, high-fat diet; wt, weight. (TIF 1021 kb) [file 13041_2018_372_MOESM1_ESM.tif]

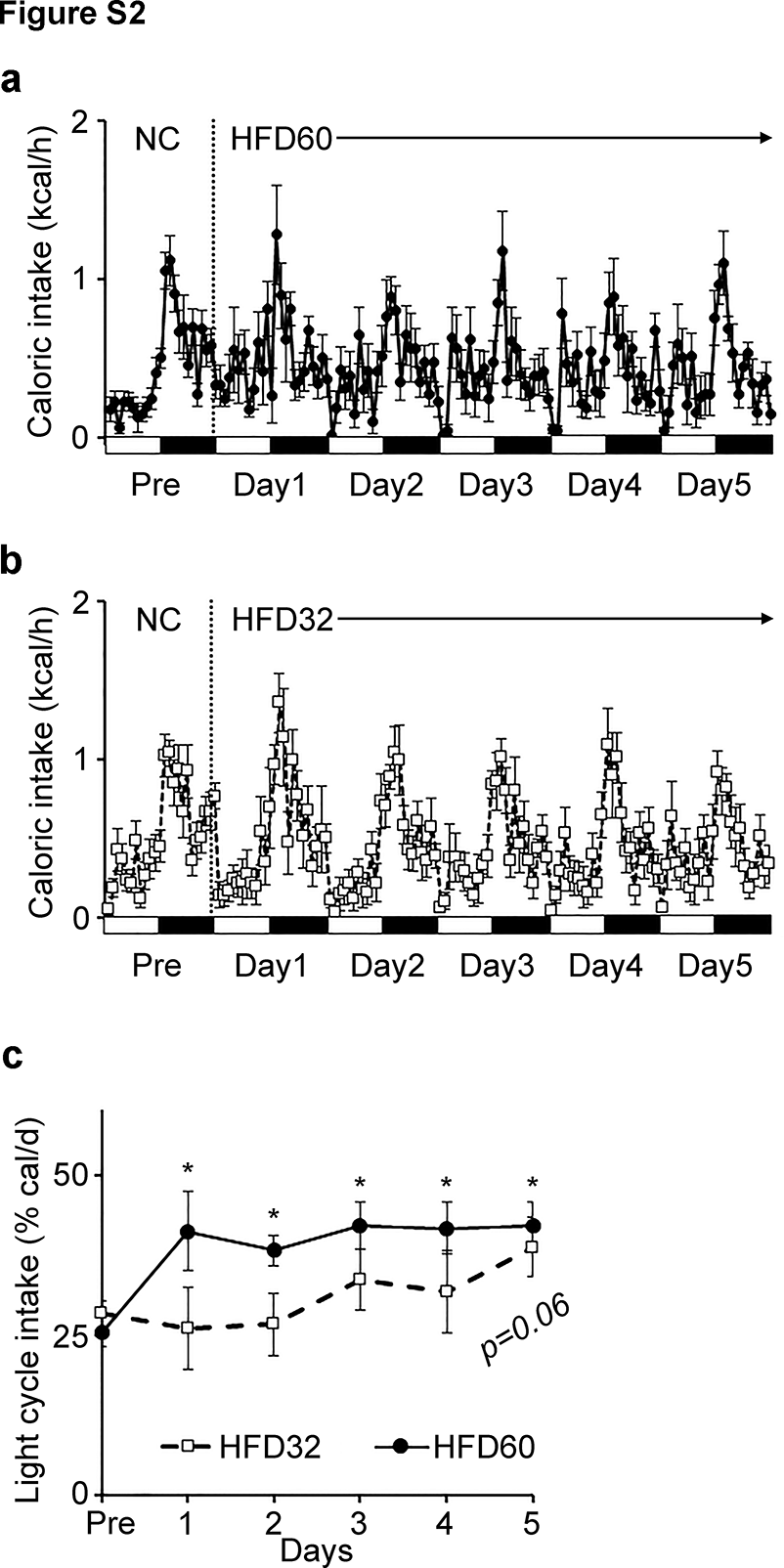

Supplement: Supplementary file 2 — Figure S2. The SFAs in HFDs disrupted feeding patterns by increasing light cycle intake. (a–c) Mice were fed one of two diets high in saturated fatty acids (SFAs); the HFD60 contained higher amounts of C16:0 and C18:0 (n = 6, black circles with solid line) than the HFD32 (n = 8, white squares with dashed line). After acclimation to FDAMS with NC, mice were fed the HFDs for 5 days. White and black bars on the X-axis correspond to the light and dark cycles, respectively. (a-b) Hourly caloric intake (1 kcal = 4.186 kJ) before (Pre) and after the diet switch from NC to (a) HFD60 or (b) HFD32. (c) The light cycle intake expressed as a percentage of the 24-h intake. Black circles with solid line: the HFD60 group; white squares with dashed line: the HFD32 group. Data are the means ± s.e.m. Statistical significance was determined with the Student’s paired t-test for comparisons to pre-diet values for each group, in c; *P < 0.05. Abbreviations: NC, normal chow; HFD, high-fat diet; FDAMS, feeding drinking, and activity monitoring system. (TIF 226 kb) [file 13041_2018_372_MOESM2_ESM.tif]

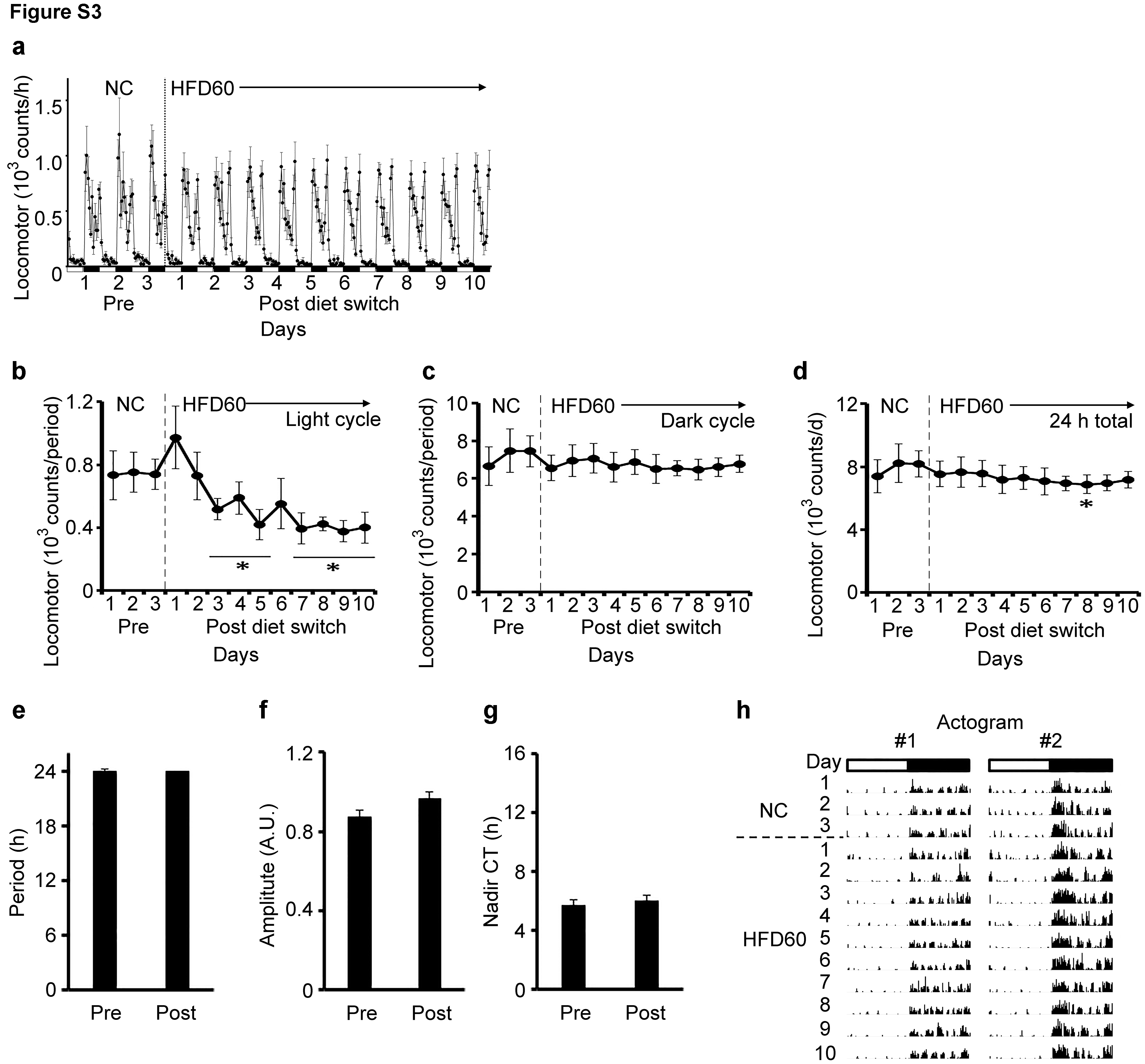

Supplement: Supplementary file 3 — Figure S3. Diet switch from NC to HFD acutely affected locomotor activity patterns in mice. Locomotor activity data for the same mice that were analyzed in Fig. 2. White and black bars on the X-axis correspond to the light and dark cycles, respectively. Vertical dashed line indicates the switch from NC (Pre) to HFD and the initiation of IP injections (Post). (a) Hourly locomotor activity over the course of the study. (b–d) Locomotor activity during (b) the light cycle, (c) the dark cycle, and (d) each 24-h period. (e-g) Cosinor analyses of the locomotor activity rhythms, including the (e) period length, (f) amplitude, and (g) nadir of the CT. (h) Actograms of two representative mice in each group. White and black bars above the traces correspond to the light and dark cycles, respectively. Data are the means ± s.e.m. Statistical significance was determined with the Student’s paired t-test, evaluated at each time point, for comparisons to the day before the diet switch (Pre in b–d, and day 0 in h). Significant differences were determined with a Student’s paired t-test for comparing data taken before and after the diet switch, in e-g. *P < 0.05. Abbreviations: A.U., arbitrary unit; CT, circadian time. (TIF 737 kb) [file 13041_2018_372_MOESM3_ESM.tif]

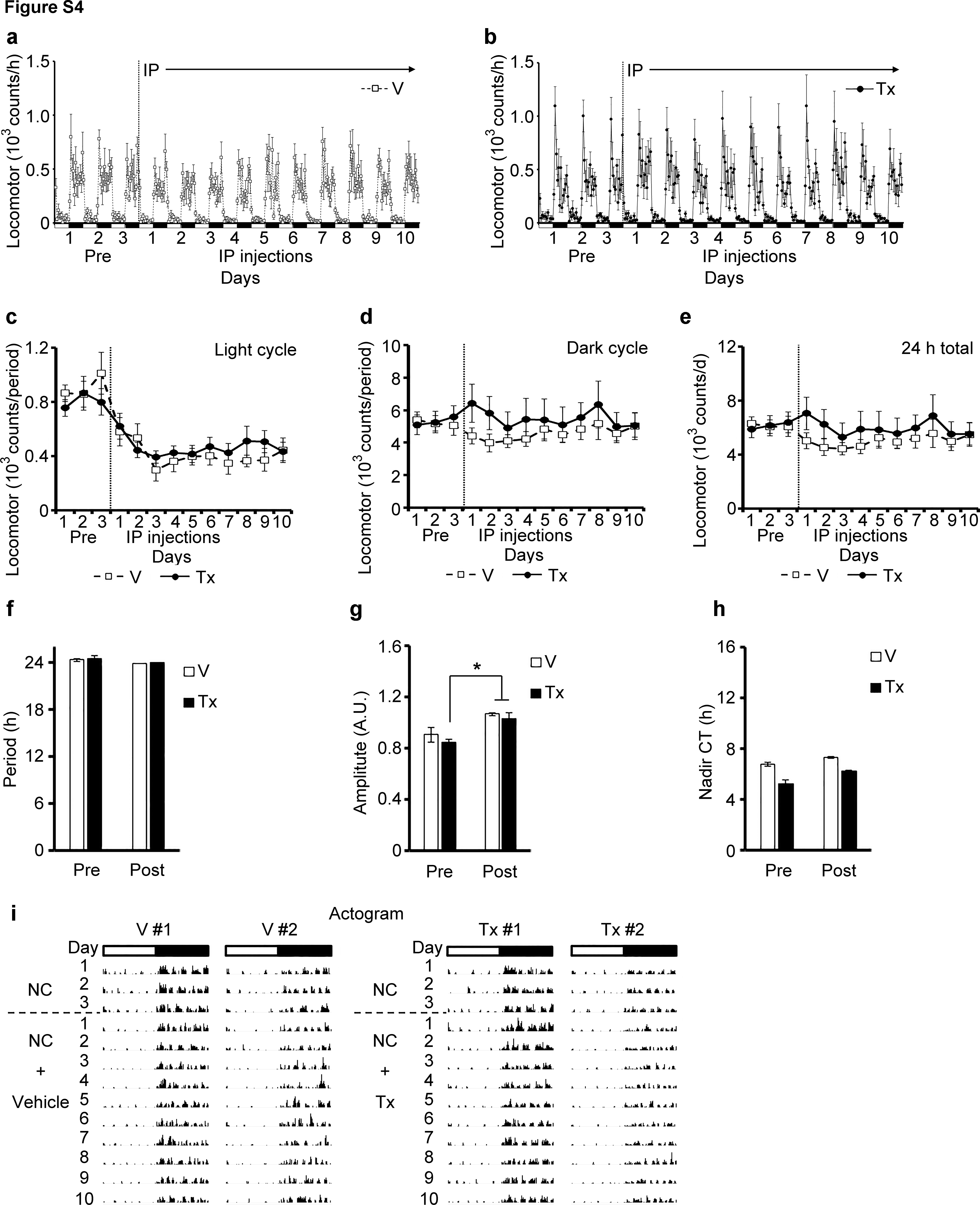

Supplement: Supplementary file 4 — Figure S4. INI-0602 did not affect locomotor activity in mice fed a normal chow diet. Locomotor activity data for the same mice that were analyzed in Fig. 5, treated with vehicle (V, n = 5, white squares with dashed line) or INI-0602 (Tx, n = 5, black circles with solid line). White and black bars on the X-axis correspond to the light and dark cycles, respectively. (a–b) Hourly locomotor activity pattern is shown before (Pre) and after (Post) initiation of intraperitoneal (IP) injections of (a) vehicle or (b) INI-0602. (c–e) Locomotor activity during (c) the light cycle, (d) the dark cycle, and (e) each 24-h period. (f–h) Cosinor analyses of the locomotor activity rhythms, including the (f) period length, (g) amplitude, and (h) nadir of the CT. (i) Actograms of two representative mice in each group. White and black bars above the traces correspond to the light and dark cycles, respectively. Data are the means ± s.e.m. Statistical significance was determined with the Student’s t-test for comparisons between the two groups at each time point, in c–e. Significant differences were evaluated with the one-way ANOVA with a post-hoc Student’s t-test and the Bonferroni correction for comparisons among groups, in f-h. *P < 0.05. Abbreviations: A.U., arbitrary unit; CT, circadian time; NC: normal chow. (TIF 1139 kb) [file 13041_2018_372_MOESM4_ESM.tif]

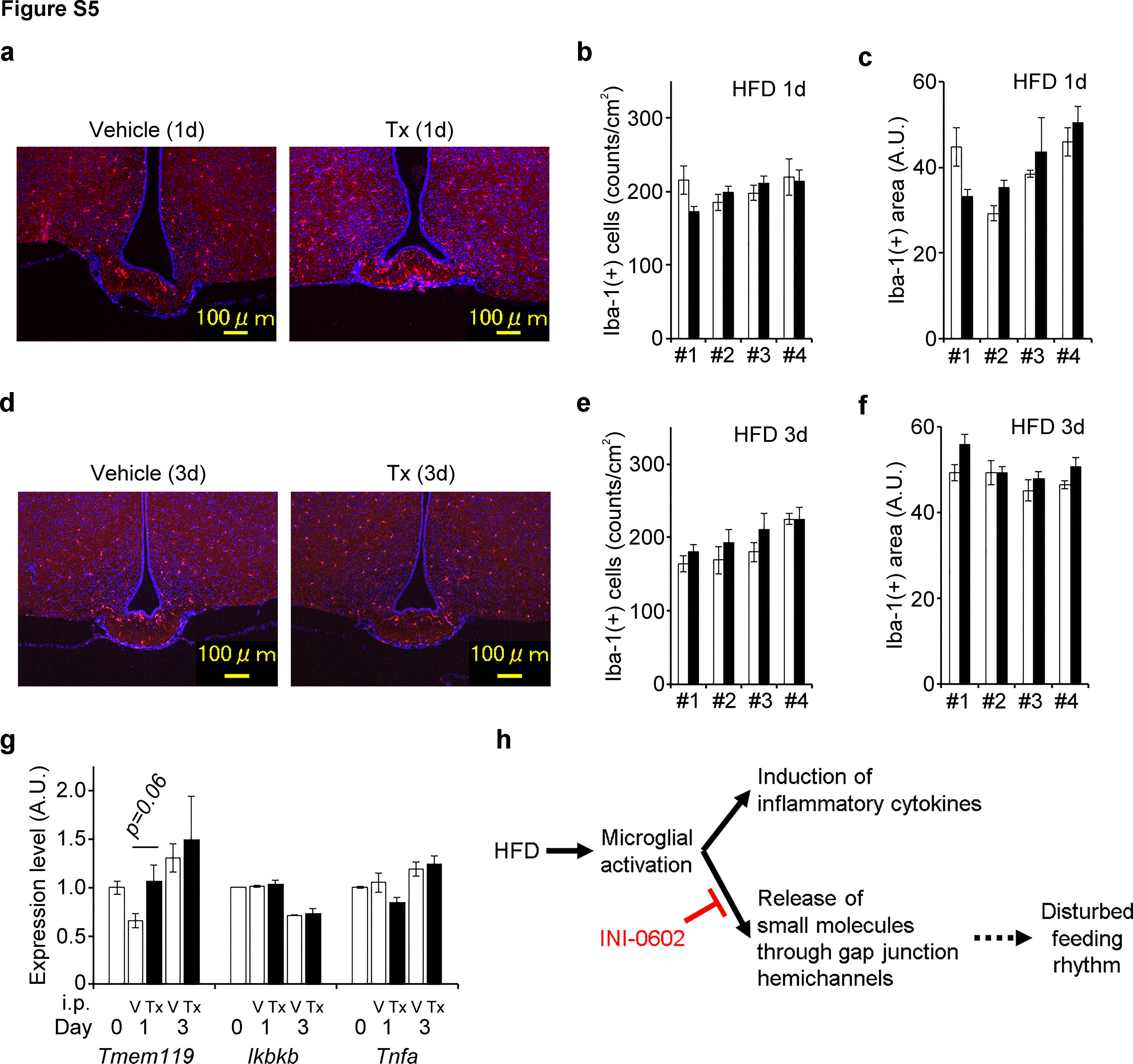

Supplement: Supplementary file 5 — Figure S5. INI-0602 did not block HFD-induced microglial activation or inflammatory cytokine expression in the hypothalamus. Mice were fed HFD for 1 or 3 days, as indicated, and they received daily intraperitoneal injections of vehicle (V, n = 3-4, white symbols) or INI-0602 (Tx, n = 4, black symbols). (a-f) Histological analyses tissue sections of the ARC of the hypothalamus. (a and d) Representative photomicrographs of ARC sections show microglia stained with anti-Iba-1 (red) and nuclei stained with DAPI (blue). Quantifications show (b and e) Iba-1 (+) cell numbers, and (c and f) Iba-1 (+) areas. Four coronal sections were analyzed per mouse. # inidicates the section number, from rostral to caudal. (g) Quantitative PCR results show hypothalamic gene expression levels measured in another cohort of mice that underwent the same treatments. (h) The proposed point of action of INI-0602 (red) in the context of HFD feeding. Data are the means ± s.e.m. Statistical significance was determined with the Student’s t-test for comparisons between groups at each time point; *P < 0.05. Abbreviations: HFD, high-fat diet; A.U., arbitrary units. (TIF 5372 kb) [file 13041_2018_372_MOESM5_ESM.tif]

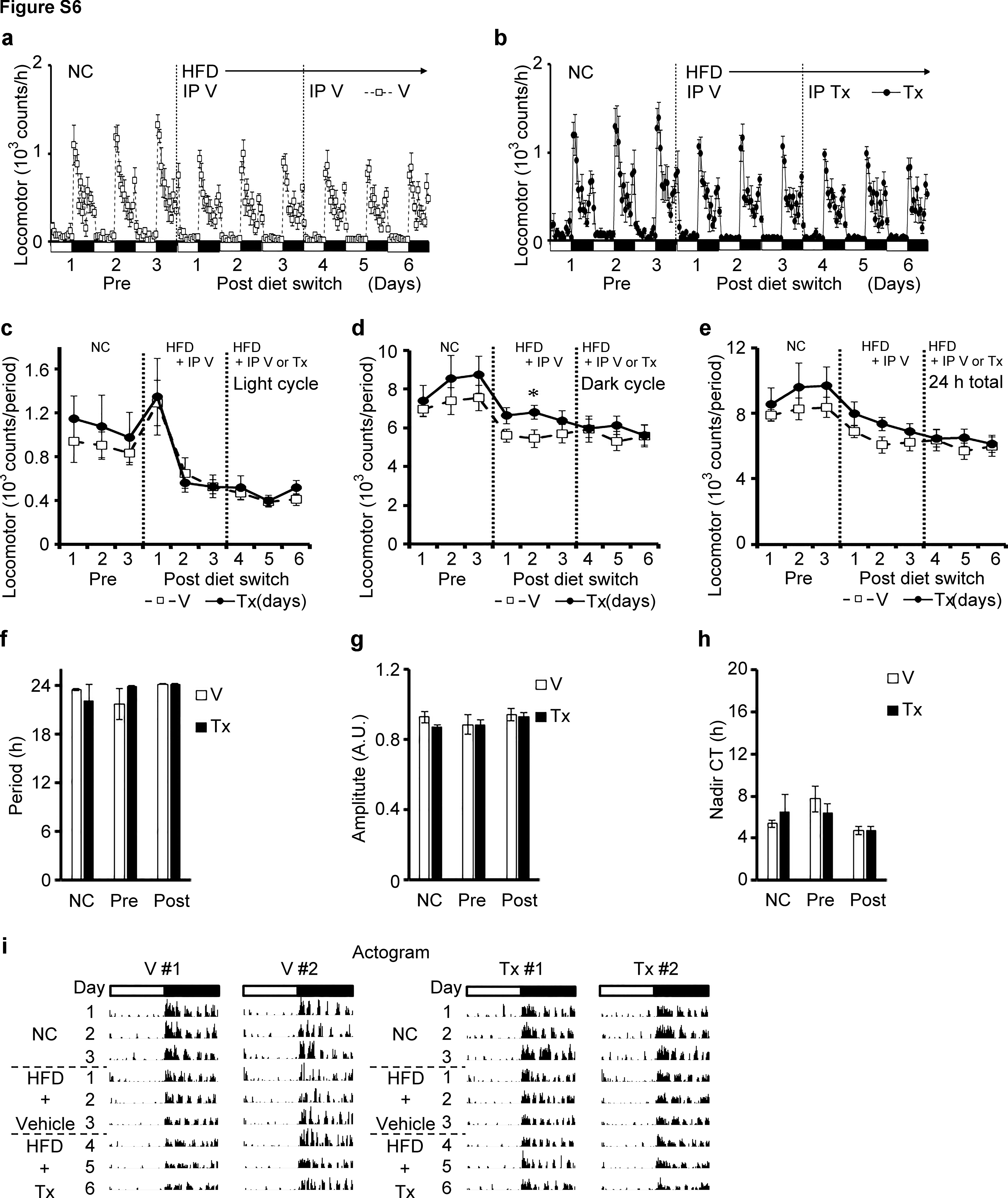

Supplement: Supplementary file 6 — Figure S6. INI-0602 given after initiating HFD feeding did not restore locomotor activity patterns in mice. Locomotor activity results for the same mice that were analyzed in Fig. 6, treated with vehicle (V, n = 6, white squares with dashed line) or INI-0602 (Tx, n = 6, black circles with solid line). White and black bars on the X-axis correspond to the light and dark cycles, respectively. Vertical dashed line indicates the switch from NC (Pre) to HFD and the initiation of IP injections (Post). (a–b) Hourly locomotor activity pattern over the course of the study in (a) the vehicle group and (b) the treated group. (c–e) Locomotor activity during (c) the light cycle, (d) the dark cycle, and (e) each 24-h period. (f–h) Cosinor analyses of the locomotor activity rhythms, including the (f) period length, (g) amplitude, and (h) nadir of the CT. (i) Actograms of two representative mice in each group. White and black bars above the traces correspond to the light and dark cycles, respectively. Data are the means ± s.e.m. Statistical significance was determined with the Student’s t-test for comparisons between groups at each time point, in c–e. Significant differences were determined with a one-way ANOVA with post-hoc Student’s t-test and the Bonferroni correction for comparisons among groups, in f-h. Abbreviations: HFD, high-fat diet; A.U., arbitrary unit; CT, circadian time. (TIF 1284 kb) [file 13041_2018_372_MOESM6_ESM.tif]
